# mRNA Targeting to Endoplasmic Reticulum Precedes Ago Protein Interaction and MicroRNA (miRNA)-mediated Translation Repression in Mammalian Cells*

**DOI:** 10.1074/jbc.C115.661868

**Published:** 2015-08-24

**Authors:** Bahnisikha Barman, Suvendra N. Bhattacharyya

**Affiliations:** From the RNA Biology Research Laboratory, Molecular Genetics Division, Council of Scientific and Industrial Research-Indian Institute of Chemical Biology, 4, Raja S C Mullick Road, Kolkata 700032, India

**Keywords:** Argonaute, endoplasmic reticulum (ER), eukaryotic translation initiation, gene expression, microRNA (miRNA), mRNA, miRNA, Polysome, Ago2, endoplasmic reticulum, translation repression

## Abstract

MicroRNA (miRNA) binds to the 3′-UTR of its target mRNAs to repress protein synthesis. Extensive research was done to understand the mechanism of miRNA-mediated repression in animal cells. Considering the progress in understanding the mechanism, information about the subcellular sites of miRNA-mediated repression is surprisingly limited. In this study, using an inducible expression system for an miRNA target message, we have delineated how a target mRNA passes through polysome association and Ago2 interaction steps on rough endoplasmic reticulum (ER) before the miRNA-mediated repression sets in. From this study, *de novo* formed target mRNA localization to the ER-bound polysomes manifested as the earliest event, which is followed by Ago2 micro-ribonucleoprotein binding, and translation repression of target message. Compartmentalization of this process to rough ER membrane ensures enrichment of miRNA-targeted messages and micro-ribonucleoprotein components on ER upon reaching a steady state.

## Teaser

**Background:** miRNA, Ago2, and target messages are enriched on ER in metazoan cells.

**Results:** Polysome association of newly formed mRNA precedes Ago2 binding on ER membrane and its translation repression.

**Conclusion:** miRNA repression process is compartmentalized in mammalian cells, and polysome association of mRNA happens before the repression.

**Significance:** This work provides a detailed understanding of the miRNA-mediated gene repression and mRNA compartmentalization in mammalian cells.

## Introduction

MicroRNAs (miRNAs)^3^ are ∼22-nucleotide-long RNAs that base-pair with the 3′-UTR of mRNAs of target genes and play an important role in gene regulation in animal and plant cells (1). miRNAs associate to a specific class of proteins known as Argonaute to form miRNPs that bind the targets to induce miRNA-mediated repression in animal cells (2). Translation of an mRNA involves different factors that are engaged in the recruitment process of ribosomal subunits and to ensure the correct initiation of protein synthesis. There are increasing evidences that miRNAs interfere with the translation initiation process (3, 4). However, whether translation of target messages is a prerequisite for miRNA-mediated inhibition is an unanswered question.

In the classical view, soluble proteins are preferentially synthesized on free cytosolic ribosomes, whereas secretory and membrane integral proteins are translated on ER-bound ribosomes. Recent investigations, however, suggest that membrane-bound ribosomes can also participate in synthesis of soluble cytosolic proteins (5). *De novo* translation initiation on ER-bound ribosomes serves as a mechanism for localizing cytosolic protein-encoding mRNAs to the ER (6).

Abundant research has already been done to understand the underlying mechanism involved in miRNA-mediated gene regulation. However, few studies have been able to identify the subcellular site where this process can take place. RNA processing bodies or P-bodies are cytoplasmic RNA granules that play a role in mRNA degradation and storage. Earlier evidences indicate localization of miRNA target messages along with the Ago2 and the cognate miRNAs to P-bodies in mammalian cells (3, 7). Recently, the role of multivesicular bodies (MVBs), organelles that control cellular miRNP trafficking, in miRNA-mediated repression process has also been reported (8). Apart from that, some evidence might be indicative of a possible role of membranous structure in miRNA function. Most miRNAs have been found to co-sediment with polyribosomes, which could be a consequence of miRNA targeting to actively translating mRNAs (9). Not only the miRNAs but also the Ago2 are associated with the actively translating polyribosomes (10, 11). Two recent studies suggested ER as the site of miRNA-mediated translation repression. In *Arabidopsis*, miRNA-mediated translation inhibition, but not mRNA cleavage, requires AMP1, an ER integral membrane protein what interacts with Argonaute1 (12). Another group has identified rough ER (rER) membrane as the central nucleation site of miRNA/siRNA loading to Ago2 proteins. They also reported that components of the RISC loading complex (RLC) co-sediment with rER membranes and identified TAR RNA-binding protein (TRBP) and protein kinase RNA activator (PACT) as key factors that anchor RISC to ER membranes in an RNA-independent manner (13).

In this study, we report preferential attachment of mRNAs having miRNA binding sites to the ER membrane. After *de novo* synthesis, the target messages get localized to the ER membrane and more specifically to the ER-bound polysomes. Here it becomes associated with the Argonaute proteins and miRNAs. Detail time course experiments have revealed that polysome localization of target messages followed by Ago2 miRNP binding on the ER membrane occurs before the miRNA-mediated translation repression sets in.

## Experimental Procedures

### 

#### 

##### Cell Culture and Transfection

HEK293 and Huh7 cells were maintained in DMEM containing 2 mm l-glutamine and 10% heat-inactivated FCS. RAW264.7 cells were maintained in RPMI 1640 medium. All cell culture reagents were from Life Technologies. All plasmid transfections were carried out with Lipofectamine 2000 (Life Technologies) using the manufacturer's protocol. For siRNA transfection, RNAiMAX was used.

##### Antibodies, Plasmids, and Reagents

Ribosomal protein S3- and calnexin-specific antibodies were from Cell Signaling Technology; anti-Ago2 antibody was from Abnova; anti-HA antibody was from Roche Applied Science; GAPDH- and β-actin-specific antibodies were from Sigma. RL-con, RL-3×bulge-let7a, and RL-3×bulge-miR-122 plasmids were kind gifts from Prof. Witold Filipowicz. Fragments encoding HMGA2 3′-UTR/HMGA2 3′-UTR mut were cloned in the XbaI and NotI sites of RL-con plasmids to get RL-HMGA2 3′-UTR/RL-HMGA2 3′-UTR mut plasmids. Tet-on advanced plasmids were from Clontech. The fragment encoding RL-3×bulge-miR-122 was re-cloned in the NheI and NotI sites of the pTRE-tight-BI vector from Clontech to get the iRL-3×bulge-miR-122 plasmid. For experiments using tetracycline-inducible constructs, induction was done using 300 ng/ml doxycycline (Sigma). Treatment with thapsigargin (Sigma) was done overnight. Expression of the HA- and N-HA-tagged versions of Ago proteins was achieved by transfecting cells with the desired plasmids obtained either from Prof. Witold Filipowicz or from Prof. Gunter Meister. SiRNA against the 3′-UTR of Ago2 was obtained from Eurogentec.

##### RNA Isolation, Northern Blotting, and Real-time PCR Analysis

Total RNA was isolated using the TRIzol reagent (Life Technologies). Northern blotting of total cellular RNA (5–10 μg) was performed as described by Pillai *et al.* (3). For detection, γ-^32^P-labeled 22-nucleotide miRCURY complementary LNA probes for let-7a (Exiqon) or complementary DNA probes for U6 snRNA were used. Phosphorimaging of the blots was performed in the Cyclone Plus storage phosphor system (PerkinElmer), and the Quant One software was used for quantification. For mRNA quantification, cDNA were prepared using random nonamers (Eurogentec reverse transcriptase core kit), and PCR was performed with gene-specific primers using the MESA GREEN qPCR Master Mix Plus (Eurogentec) following the manufacturer's protocol.

##### Immunoprecipitation and Western Blotting

Immunoprecipitation of tagged AGO2 was done as per published protocols (14, 15). Briefly, cell homogenate obtained with 0.5% Triton X-100 and 0.5% sodium deoxycholate was cleared by centrifugation before being used for immunoprecipitation using the specific antibodies bound to protein G-agarose beads.

##### Cell Fractionation and Polysome Isolation

For digitonin fractionation, 1 × 10^6^ cells were lysed in the digitonin lysis buffer (10 mm Tris, pH 7.5, 25 mm KCl, 5 mm MgCl_2_, 1 mm CaCl_2_, 5 mm vanadyl ribonucleoside complex (Sigma), 1 mm DTT, 1× protease inhibitor cocktail (Roche Applied Science), and 50 μg/ml digitonin (Calbiochem)) for 10 min on ice. The membrane fraction was collected by spinning at 2,500 × *g* for 5 min followed by washing the membrane pellet with the same lysis buffer without digitonin. RNA isolation from the each fractions was done using acidic phenol:chloroform (5:1) or TRIzol LS (Invitrogen).

For microsome isolation, HEK293 cells were resuspended in 1× hypotonic buffer (10 mm HEPES, pH 7.8, 1 mm EGTA, 25 mm KCl) equivalent to three times the packed cell volume and incubated for 20 min on ice. The cells were spun down and resuspended in 1× isotonic buffer (10 mm HEPES, pH 7.8, 1 mm EGTA, 25 mm KCl, and 250 mm sucrose) twice the packed cell volume and homogenized. The cell lysate was precleared at 1,000 × *g* for 10 min, followed by a 12,000 × *g* spin for 10 min to remove the mitochondrial fraction. The post-mitochondrial supernatant was incubated for 15–20 min with 8 mm CaCl_2_, followed by centrifugation at 8,000 × *g* for 10 min to obtain the microsomal pellet (16, 17).

For KCl-puromycin extraction, a microsome pellet was resuspended in KCl- and puromycin-containing buffer (50 mm Tris-HCl, pH 7.5, 250 mm sucrose, 2 mm MgCl_2_, 500 mm KCl, 1 mm puromycin, 1× protease inhibitor cocktail (Roche Applied Science), and 5 mm vanadyl ribonucleoside complex (Sigma)) and incubated for 15 min at 37 °C. The extracts were centrifuged at 100,000 × *g* for 1 h to separate the ribosomal fraction as supernatants and the non-ribosomal membrane fraction as pellet as described (17, 18).

Cell fractionation using OptiPrep density gradient centrifugation and total polysome isolation were carried out as described previously (19). All graphs and statistical analyses were done in GraphPad Prism 5.00 (GraphPad, San Diego, CA). Nonparametric paired *t* test was used for analysis, and *p* values were determined. Error bars indicate mean ± S.E.

## Results

### 

#### 

##### Association of miRNA, Ago2, and Target Messages with ER-attached Polysomes

**FIGURE 1.**
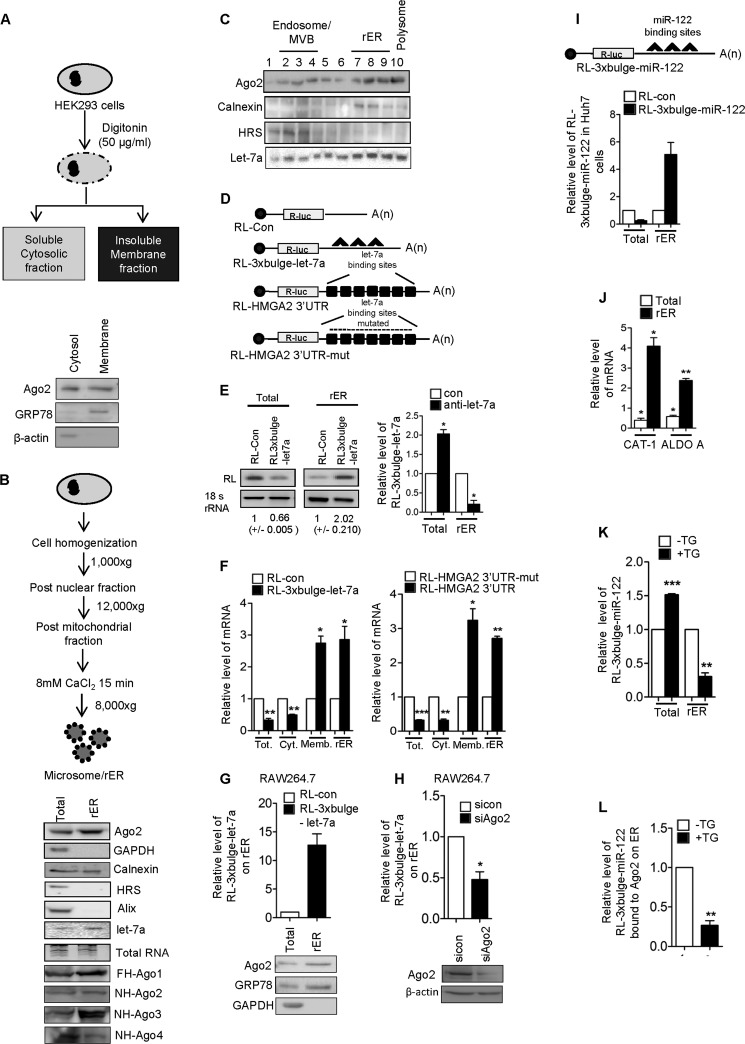
**Enrichment of miRNA and targeted messages on ER in mammalian cells.** *A*, a schematic representation of cell permeabilization with digitonin. Ago2 was immunoblotted in the cytosolic and membrane fractions after cell fractionation with digitonin; GRP78 and GAPDH served as the membrane and cytosolic markers, respectively. *B*, a scheme for isolation of rER or microsomes. Western and Northern blots were done for Ago2 and let-7a, respectively, for total and isolated microsomal (rER) fractions. Calnexin served as a marker of endoplasmic reticulum. The absence of hepatocyte growth factor-regulated tyrosine kinase substrate (*HRS*) and Alix (MVB markers) and GAPDH (cytosolic marker) confirms the purity of isolated microsomes. Fractionation data of cells expressing tagged versions of Ago proteins are shown in the *lower panels. C*, levels of Ago2 and let-7a in different fractions of an iodixanol density gradient (OptiPrep gradient) (3–30%) of HEK293 cell lysate. Levels of markers (calnexin and HRS) in Western blot data confirmed separation of individual organelles on the gradients. *Frac. No.*, fraction number. *D*, *left panel*, plasmid constructs used for following the miRNA target distribution in mammalian cells. *E*, semi-quantitative RT-PCR analysis of RL-con or RL-3×bulge-let-7a in total cell lysates and microsomal fraction. *Right panel*, effect of anti-let-7a treatment on cellular and rER-associated target mRNA levels in HEK293 cells. *F*, relative levels of exogenously expressed RL-3×bulge-let7-a or RL-HMGA2 3′-UTR were plotted in different fractions of HEK293 cells after quantitative RT-PCR. Real-time values obtained from RL-con or RL-HMGA2 3′-UTR-mut (with mutated let-7a binding sites) were taken as 1. *G*, enrichment of let-7a target message and Ago2 on rER isolated from RAW264.7 cells. *H*, effect of Ago2 depletion on let-7a target message association with rER. *I*, enrichment of target mRNAs in microsomal fraction in the presence of the miRNA. Quantitative analysis of RL-con and RL-3×bulge-miR-122 in total and ER associated RNA in Huh7 cells expressing miR-122 was performed. *J*, induced expression of miR-122 resulted in enhanced association of miR-122 targets in HEK293 cells. Levels of CAT-1 and aldolase, two well characterized miR-122 target messages, were followed in the total and ER fraction after 24 h of miR-122 induction in HEK293 cells. In each case, the level of mRNA in 0 h was taken as 1. *K*, effect of TG on RL-3×bulge-miR-122 localization on the ER membrane. Total and ER-bound RL-3×bulge-miR-122 level were measured and plotted. Total and ER-bound RL-3×bulge-miR-122 level were measured by RT-qPCR and plotted when cells were treated with TG (5 μm) or dimethyl sulfoxide overnight. *L*, effect of TG on RL-3×bulge-miR-122 association with ER-bound Ago2. Ago2 was immunoprecipitated from the isolated ER fraction of FLAG-HA-Ago2 (FA-Ago2) stable HEK293 cells treated with TG. Ago2-associated RL-3×bulge-miR-122 was quantified and plotted after normalization with Ago2. In all RT-qPCR experiments, 18s rRNA served as the endogenous control. For estimation of RNA and proteins, we used cell-equivalent amounts in individual reactions. RT-qPCR results from three independent experiments ± S.D. are shown, and the values of control are normalized to 1 (*, *p* < 0.05; **, *p* < 0.01; ***, *p* < 0.001).

We wanted to identify the cytoplasmic compartments with which miRNAs, Ago proteins, and target messages are associated with in mammalian cells. To do so, we treated HEK293 human cells with digitonin, a detergent that, when used at a lower concentration, preferentially permeabilizes plasma membranes with higher cholesterol contents while leaving low cholesterol-containing internal organelles intact. Digitonin treatment of HEK293 cells yielded a detergent-insoluble ER marker protein GRP78-positive membrane fraction, and a β-actin-positive cytoplasmic fraction. We found enrichment of Ago2 in the digitonin-insoluble membrane fraction (Fig. 1*A*). As an alternative approach, we have isolated rER from isotonic lysates of HEK293 cells. The isolated rER was positive for the ER marker calnexin and negative for MVBs and the late endosomal marker Alix. The isolated rER or microsomes were found to be enriched for both Ago2 and miRNA (Fig. 1*B*). To confirm the association of miRNA and Ago2 protein with rER and associated structures, we fractionated isotonic HEK293 cell lysates on a 3–30% OptiPrep gradient and analyzed the individual fractions. We observed that the majority of Ago2 and let-7a miRNA accumulated with the ER-positive fractions (Fig. 1*C*). With isolated RNAs, we detected that enhanced accumulation of let-7a repressed target message RL-3×bulge-let-7a with rER (Fig. 1, *D* and *E*). Individually tested, two different let-7a reporter mRNAs also showed significant enhancement in their association with the rER and digitonin-insoluble membrane fractions (Fig. 1, *E* and *F*). We also found that almost 71% of the total mRNA transcripts were ER-bound for let-7a target message when only 29% of total non-target mRNAs are ER-attached. More than 38% of let-7a miRNAs are rER-attached. miRNA dependence of target message accumulation on rER in the steady state was confirmed in cells inactivated for let-7a where the corresponding target message failed to accumulate on rER (Fig. 1*E*). This phenomenon is not HEK293- or let-7a miRNA-specific, and we documented a similar observation for a let-7a reporter in RAW264.7 mouse monocyte/macrophage cells and also for an miR-122 reporter in Huh7 cells expressing miR-122 (Fig. 1, *G–I*). The Ago2 dependence of the phenomenon was noted when depletion of the cellular Ago2 level caused decreased association of miRNA targets with rER in RAW264.7 cells (Fig. 1*H*). With ectopic expression of miR-122 in HEK293 cells, we could also document membrane association of two endogenous miR-122 targets, aldolase and CAT-1 mRNAs, in HEK293 cells (Fig. 1*J*). Therefore, both miRNPs and their targets could accumulate on rER in mammalian cells.

The ER association of target message is dependent on ER function. Thapsigargin (TG), an inducer of ER stress, has been shown previously to affect protein translation (20). Consistent with these observations, we documented a gross reduction in association of miRNA-targeted messages with rER upon application of TG, although no major effect was seen on the total cellular content of target mRNAs (Fig. 1*K*). Importantly, our results also showed the compromised binding of mRNA with Ago2. (Fig. 1*L*).

**FIGURE 2.**
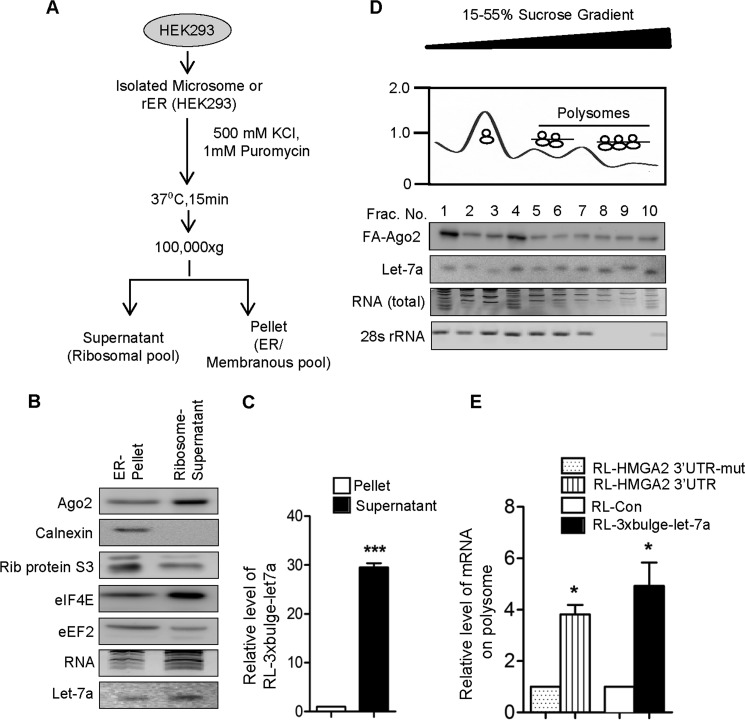
**miRNA-targeted messages are bound to ER-attached polysomes.** *A*, schematic representation of the isolation of polysomes attached to the rER. *B*, levels of Ago2 and miRNA in KCl-puromycin extract or rER. Ribosomal protein S3 (*Rib. protein S3*), eIF4E, eEF2, and ER integral membrane protein calnexin were used to monitor the extraction of rER-attached ribosome without an effect in ER integrity. *C*, level of miRNA-targeted message in KCl-puromycin extract and residual pellet fraction. *D*, 15–55% sucrose density gradient fractions of isolated microsomes of HEK293 cells expressing FLAG-HA-Ago2 (FA-Ago2). Endogenous let-7a level and FH-Ago2 were detected in different fractions by Northern and Western blot, respectively. The relative presence of 28s RNA was determined in individual fractions by semi-quantitative RT-PCR, and absorptions at 260 nm were plotted. Positions of the fractions with polysomes are marked. *Frac. No.*, fraction number. *E*, levels of RL-HMGA2-3′-UTR or RL-3×bulge-let-7a mRNAs were quantified by RT-qPCR in isolated polysomal fractions in HEK293 cells. RL-HMGA2 3′-UTR-mut or RL-con were taken as a control. In all RT-qPCR experiments, 18s rRNA served as the endogenous control. RT-qPCR results from three independent experiments ± S.D. are shown, and the values of control are normalized to 1 (*, *p* < 0.05; **, *p* < 0.01; ***, *p* < 0.001).

To identify and characterize the rER-associated miRNPs and target messages, we treated rER with KCl and puromycin to extract the polysomes associated with rER. Upon treatment, we found the majority of miRNA, Ago2, and target messages, along with ribosomal components, in the supernatant (Fig. 2, *A–C*). Polysome association of the miRNA and Ago2 on rER was further validated when the extract of rER was analyzed on a 15–55% sucrose density where a large fraction of Ago2 and the majority of let-7a were found to be with the polysomal fraction (Fig. 2*D*). Interestingly, the let-7a targets also showed enhanced accumulation in the rER-attached polysomes in HEK293 cells (Fig. 2*E*).

##### Ago2 Binding of mRNA on rER Precedes Repression

**FIGURE 3.**
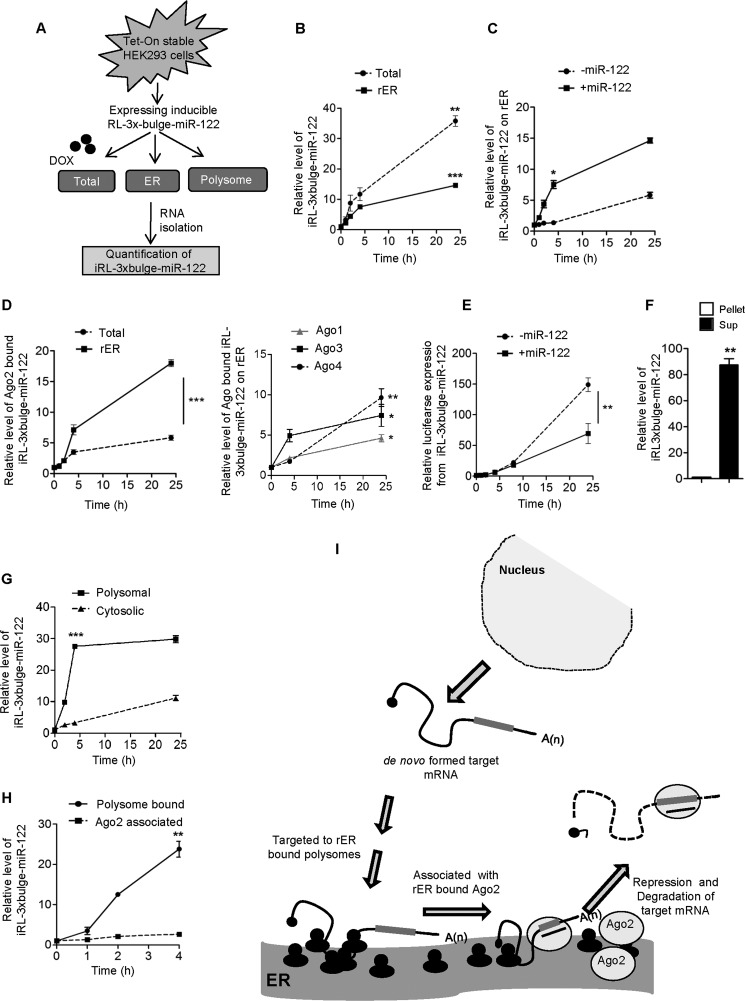
**Polysome targeting precedes Ago2 and miRNA interaction and repression of target mRNAs on ER.** *A*, schematic representation of the experiments. *DOX*, doxycycline. *B*, time-dependent changes in the levels of RL-3×bulge-miR-122 after 0, 1, 2, 4, and 24 h of induction in the total and rER-associated fractions. *C*, relative level of *de novo* formed RL-3×bulge-miR-122 associated with rER in the presence and absence of miR-122 in HEK293 cells. Levels of RL-3×bulge-miR-122 on the ER membrane were measured and plotted when cells were transfected with miR-122-expressing plasmid or control vector. *D*, time-dependent increase in Ago2 association of RL-3×bulge-miR-122. Tet-On stable HEK293 cells were transiently transfected with FA-Ago2, pre-miR-122, and Tet-inducible RL-3×bulge-miR-122-expressing plasmids. Ago2 was immunoprecipitated from either total cell lysates or microsomes after different induction times. Ago2-associated RL-3×bulge-miR-122 was measured and plotted (*left panel*). Similar experiments were done with other Ago proteins, and relative quantifications of associated target messages were performed and plotted. *E*, time-dependent expression of RL-3×bulge-miR-122 in the presence or absence of miR-122. RL-3×bulge-miR-122 expression was measured after 0, 1, 2, 4, 8, and 24 h of induction by luciferase assay and plotted the relative expression level in both cases. Firefly luciferase (FF) encoded from a co-transfected plasmid serves as the endogenous control. *F*, co-extraction of *de novo* formed RL-3×bulge-miR-122 with ER-attached ribosomes. RL-3×bulge-miR-122 level was quantified in KCl-puromycin extracts of microsomal fraction after 4 h of induction and plotted. RL-3×bulge-miR-122 in the non-extracted part served as the control. *Sup.*, supernatant. *G*, time course of RL-3×bulge-miR-122 associated with polysomes and in the cytosol in the presence of miR-122. After 0, 2, 4, and 24 h of induction, RL-3×bulge-miR-122 levels were quantified and plotted. HEK293 cells were transfected with miR-122-expressing plasmids. *H*, quantification of time-dependent Ago2 and polysome association of RL-3×bulge-miR-122 after induction. mRNA was quantitatively measured in immunoprecipitated Ago2 from Tet-On stable HEK293 cells and in isolated polysomes after 0, 1, 2, and 4 h of induction and then plotted. *I*, model for the sequential events of target mRNA binding to polysomes, followed by its interaction with Ago2 on the ER and repression. In *B–H*, all real-time values were normalized by endogenous 18s rRNA. In all cases, values of 0 h were taken as 1. RT-qPCR and luciferase results from at least three independent experiments ± S.D. are shown, and the values of control are normalized to 1 (*, *p* < 0.05; **, *p* < 0.01; ***, *p* < 0.001).

From the observations documented in the previous section, association of target messages along with Ago2 on the ER-bound ribosomes was evident. To investigate whether ER targeting is a prerequisite for miRNA-mediated repression, we generated a tetracycline-induced expression system where expression of RL-3×bulge-miR-122, an miR-122 reporter mRNA, is dependent on doxycycline (Fig. 3*A*). As expected, upon application of the drug, we found a sharp increase of reporter mRNA level with time in the total cell lysates of HEK293 cells. Interestingly, on the ER membrane, *de novo* formed RL-3×bulge-miR-122 also accumulates against time (Fig. 3*B*). Targeting of RL reporter to rER was much faster in HEK293 cells in the presence of miR-122. However, Ago2 binding kinetics of miR-122 in the inducible expression system was much faster for pools of Ago2 associated with rER as compared with its binding rate with total Ago2 (Fig. 3, *C* and *D*). Other Ago proteins also showed time-dependent increase in association with *de novo* synthesized target messages on rER membranes (Fig. 3*D*). Does this indicate that *de novo* formed ER-bound target messages are already repressed? To answer this, we measured the expression level of the RL-3×bulge-miR-122 reporter against time in the presence and absence of miR-122 and found translation repression only after 8 h when targeting and binding of mRNA to ER-attached Ago2/miRNPs have already occurred (Fig. 3*E*). Therefore, Ago2 binding of *de novo* synthesized mRNAs on the ER precedes the miRNA-mediated repression process in mammalian cells.

In the steady state, we found enrichment of induced mRNA within the ribosomal pool associated with rER (Fig. 3*F*). Do *de novo* formed target messages attach to the ribosomal pool of the ER membrane even before Ago2 or miRNA interaction? To determine that, we measured the level of RL-3×bulge-miR-122 associated with polysomes after induction and found rapid association of this level with the ribosomal pool of ER within 4 h, which was much faster than what we observed with cytoplasmic polysomes (Fig. 3*G*). To find out the sequence of events, we measured the induced RL-3×bulge-miR-122 level within a short time window after the induction of its transcription and found, within 4 h, a visible enrichment of this mRNA on polysomes. However, during the same time, we did not find any significant increase in its interaction with the Ago2 (Fig. 3*H*).

Taken together our data suggest the targeting of an mRNA bearing an miRNA binding site on its 3′-UTR to the ER membrane-bound polysomes after its synthesis and before its interaction with Ago2 and repression. Our observation thus has helped to elucidate the exact sequence of this *de novo* target message localization to the subcellular compartments before repression. These data revealed that the polysome localization was the initial event for a *de novo* formed target mRNA. Ago2 protein binding on the ER membrane would be the next event, followed by the miRNA-driven repression of the target messages.

## Discussion

It was predicted and shown earlier that translation repression by miRNA happens at the translation initiation step, followed by the degradation of the miRNA-targeted mRNAs in mammalian cells (21, 22). However, the exact sequence of events in the miRNA-mediated repression process was not known. Here we have documented that in human cells, miRNA target messages are getting enriched on the ER membrane, along with Ago2 and cognate miRNAs. The ER membrane acts as the site where a *de novo* synthesized target mRNA becomes associated with miRNA and Ago2 proteins. These target messages are preferentially co-extracted with the membrane-attached ribosomes. We also showed evidence that *de novo* formed target mRNAs destined to bind to membrane bound to the polysomes first, followed by Ago2/miRNP association on the ER membrane and repression.

Our study recognized the ER membrane as the primary site of target mRNA binding with Ago2 proteins. Conventionally, the ER is the established site for translation of mRNA-encoding membrane-bound or secretory proteins. These mRNAs are recruited to the ER via a signal recognition particle in a translation-dependent manner. However, many studies report the puzzling observations that soluble cytosolic proteins are translated on membrane-bound ribosomes and that soluble protein-coding mRNA visits ER in a signal sequence- and translation-independent manner (5, 23). A few other studies have proposed that the ER membrane acts as the site of translation regulation. Most miRNAs are associated with actively translating mRNAs and co-sedimented with polyribosomes (9). Ago2 also co-sediments with polyribosomes (10). In our study, we ascertained that the ER membrane acts as the site of target message assembly. Target mRNA localization to the membrane-bound polysome initializes the sequence, which is followed by Ago2 binding on ER membrane. Target mRNA binding with ER-bound Ago2 leads to translation repression as an ultimate event on the membrane.

Additionally, we have been able to conclusively show that translation of mRNA is the default step that precedes miRNA-mediated repression. This is useful information toward understanding the mechanism of miRNA-mediated gene regulation in mammalian cells. The advantage of compartmentalization of the repression process is not apparent, but it could ensure rapid shuttling of the messages to the storage- or degradation-specific compartments in successive steps in mammalian cells.

## Author Contributions

S. N. B. conceived and coordinated the study, analyzed the experiments, and wrote the paper. B. B. performed and analyzed the experiments.

## Footnotes

3 The abbreviations used are:

miRNA: microRNA
miRNP: micro-ribonucleoprotein
miR: miRNA
ER: endoplasmic reticulum
rER: rough ER
MVB: multivesicular body
RISC: RNA-induced silencing complex
RT-qPCR: RT-quantitative PCR
TG: thapsigargin
mut: mutant.
